# Glycated albumin and its variability as an indicator of cardiovascular autonomic neuropathy development in type 2 diabetic patients

**DOI:** 10.1186/s12933-017-0619-2

**Published:** 2017-10-10

**Authors:** Ji Eun Jun, Seung-Eun Lee, You-Bin Lee, Ji Yeon Ahn, Gyuri Kim, Sang-Man Jin, Kyu Yeon Hur, Moon-Kyu Lee, Jae Hyeon Kim

**Affiliations:** 1 Division of Endocrinology and Metabolism, Department of Internal Medicine, Samsung Medical Center, Sungkyunkwan University School of Medicine, 81 Irwon-ro, Gangnam-gu, Seoul, 06351 Republic of Korea; 2 Department of Clinical Research Design & Evaluation, SAIHST, Sungkyunkwan University, 81 Irwon-ro, Gangnam-gu, Seoul, Republic of Korea

**Keywords:** Glycated albumin, Cardiovascular autonomic neuropathy, Glycemic variability, Type 2 diabetes

## Abstract

**Background:**

We investigated whether glycated albumin (GA) and its variability are associated with cardiovascular autonomic neuropathy (CAN) and further compared their associations with glycated hemoglobin (HbA1c).

**Methods:**

This retrospective longitudinal study included 498 type 2 diabetic patients without CAN. CAN was defined as at least two abnormal results in parasympathetic tests or presence of orthostatic hypotension. The mean, standard deviation (SD), and coefficient of variance (CV) were calculated from consecutively measured GA (median 7 times) and HbA1c levels (median 8 times) over 2 years. Logistic regression analysis was used to compare the associations between CAN and GA- or HbA1c-related parameters. Receiver operating characteristic (ROC) curve analysis was used to compare the predictive power for CAN between GA- and HbA1c-related parameters.

**Results:**

A total of 53 subjects (10.6%) developed CAN over 2 years. The mean, SD, and CV of GA or HbA1c were significantly higher in subjects with CAN. Higher mean GA and GA variability were associated with the risk of developing CAN, independent of conventional risk factors and HbA1c. In ROC curve analysis, the SD and CV of GA showed higher predictive value for CAN compared to the SD and CV of HbA1c, whereas the predictive value of mean GA did not differ from that of mean HbA1c. The mean, SD, and CV of GA showed additive predictive power to detect CAN development along with mean HbA1c.

**Conclusions:**

Higher serum GA and its variability are significantly associated with the risk of developing CAN. Serum GA might be a useful indicator for diabetic complications and can enhance HbA1c’s modest clinical prediction for CAN.

**Electronic supplementary material:**

The online version of this article (doi:10.1186/s12933-017-0619-2) contains supplementary material, which is available to authorized users.

## Background

Cardiovascular autonomic neuropathy (CAN) is a common and clinically significant diabetic complication that causes cardiovascular disease (CVD), mortality, and morbidity in diabetic patients [1]. Both chronic hyperglycemia and glucose fluctuation are important causes of the initiation of the pathogenic process of CAN, as they result in increased oxidative stress, which induces direct neural damage and indirect neuronal ischemia from endothelial dysfunction [2, 3].

Although glycated hemoglobin (HbA1c) is the gold-standard parameter for glycemic control, emerging evidence has shown that glycated albumin (GA) is a useful indicator of glycemic control in subjects with hematologic disorders such as anemia, chronic kidney disease, or hemorrhage, which can affect HbA1c level [4]. Serum GA provides a more accurate assessment of recent glycemic change due to the short half-life (2-3 weeks) of albumin [5], and it is more strongly associated with glucose excursions, whereas HbA1c reflects average glucose level rather than glycemic variability (GV) [6, 7]. GA actually correlates with diabetic complications such as retinopathy progression [8], chronic kidney disease [9], peripheral neuropathy [10], and even CVD [11, 12].

Most of the studies about GA and diabetic complications used GA measured at one time point, which does not reflect the long-term glycemic state. Although HbA1c variability as a long-term GV parameter has been associated with diabetic complications independent of mean HbA1c [13–15], there have been few studies of GA variability and diabetic complications. This study aims to investigate CAN and its relationship to both mean GA level and GA fluctuation in type 2 diabetic patients compared to mean HbA1c and HbA1c fluctuation.

## Methods

### Study subjects

A total of 1019 adult type 2 diabetes mellitus (T2DM) patients (age ≥ 18 years) who underwent CAN testing in the outpatient diabetes clinic of Samsung Medical Center in Seoul, Republic of Korea, at least twice at 2-year intervals were screened from September 2011 to March 2017. T2DM was diagnosed based on the 2017 American Diabetes Association guidelines. The exclusion criteria were as follows (Additional file 1: Figure S1): clinical diagnosis of type 1 diabetes (*n* = 111); history of CVD including any form of coronary artery disease, congestive heart failure, arrhythmia, valvular disease, or ischemic stroke (*n* = 81); history of liver disease or abnormal liver function test at baseline (*n* = 38); any malignancy upon treatment (*n* = 56); pancreatitis or pancreatectomy (*n* = 13); any transplantation (*n* = 10); hyper- or hypothyroidism (*n* = 17); history of chronic kidney disease or estimated glomerular filtration rate (eGFR) calculated by the CPD-EPI equation ≤ 60 ml/min/1.73 m^2^ (*n* = 54); CAN diagnosis at the time of enrollment (*n* = 393); follow-up of less than 2 years (*n* = 153); missing serial measurements of GA or HbA1c for 2 years (*n* = 82); other missing clinical variables (*n* = 11).

Finally, 498 subjects (299 men and 199 women) who were never diagnosed with CAN were enrolled in the study. The study protocol was approved by the Institutional Review Board of Samsung Medical Center.

### Demographic and clinical assessment

Patient medical history, medication, smoking, and alcohol drinking status data were extracted from electronic medical records for the same day that the first CAN test was administered. HbA1c level was measured by high-performance liquid chromatography, using a VARIANT II TURBO analyzer (Bio-Rad Laboratories, Hercules, CA, USA). Serum GA level was measured by an enzymatic method using a Lucica GA-L kit (Asahi Kasei Pharma Corporation, Tokyo, Japan). The reference interval for HbA1c was 4.0–6.0%, and that for GA was 11.0–16.0%. Serum GA (median number of measurements 7, interquartile range [IQR] 6–8) and HbA1c levels (median number of measurements 8, IQR 7–8) were consecutively measured every three to 6 months for 2 years. Mean values were calculated for GA and HbA1c levels measured during the 2 years after recruitment to the day of the CAN test. The variabilities of GA and HbA1c were evaluated using the intrapersonal standard deviation (SD) and coefficient of variance (CV) of the serial measurements of GA and HbA1c. Because few visits result in apparently larger SD than many visits, the adjusted SD was defined as the SD divided by [n/(n − 1)]^0.5^, where n is the number of GA or HbA1c measurements [16], and was used in the analyses instead of the unadjusted SD.

After an 8-h overnight fast, plasma glucose level was measured using the glucose oxidase method, and serum C-peptide level was measured in duplicate with immunoradiometric assays (Beckman Coulter, Fullerton, CA, USA). Lipid profiles were assayed using a Hitachi 7600 auto analyzer (Hitachi Instruments Service, Tokyo, Japan).

### Assessment of CAN

Cardiovascular tests based on heart rate response and orthostatic hypotension are an essential and irreplaceable component of CAN diagnosis [17–19]. Patients were advised to avoid strenuous physical exercise, tobacco, and alcohol in the 24-h preceding the test and to avoid coffee and food for at least 3-h prior to the test. Medications such as anti-histamines, anti-depressants, and β-blockers were withheld for 12-h prior to the test.

Three tests mainly assess parasympathetic function: heart rate variability (HRV) to deep breathing (exhalation: inhalation ratio), to standing (30:15 ratio), and to the Valsalva maneuver (Valsalva ratio); blood pressure response to standing is used to assess sympathetic function. The heart rates responses were assessed automatically from electrocardiography recordings using the DICAN evaluation system (Medicore Co., Ltd., Seoul, Korea). Change in systolic blood pressure was obtained from the difference between the value measured in the supine position and the value measured 60 s after standing.

Values below the lower limit of an age-specific reference range suggested in our previous study were considered abnormal [13]. CAN was defined as the presence of two or more abnormal results among the three parasympathetic tests (definite CAN) or presence of orthostatic hypotension (severe CAN) [20].

### Statistical analysis

All statistical analyses were performed using SPSS version 24.0 software (Chicago, IL, USA) and STATA/SE version 14.0 (StataCorp LP, College Station, TX, USA). Data are expressed as mean ± SD, median with IQR, and number (%). Student’s *t* test and the Mann–Whitney *U* test were used to assess the differences in continuous variables between two groups, while the Chi square test was used for categorical variables. Scatter plots and linear analyses were used to evaluate the correlations between mean GA and mean HbA1c as well as their mean values and GV parameters. Binary logistic regression analyses assessed the associations between CAN and various glycemic parameters. Multivariate logistic regression analysis was adjusted for covariates that were significant in univariate analysis and those that were conventional risk factor of CAN with the enter method, along with each glycemic parameter as an independent variable. A variance inflation factor > 5.0 was used as an indicator of multicollinearity. The area under the curve (AUC) was obtained from receiver operating characteristic (ROC) curve analyses to compare the predictive values between GA- and HbA1c-related parameters. ROC analyses were also adjusted using the enter method. The level of significance was set at *P* < 0.05 in two-tailed tests.

## Results

### Baseline characteristics of all patients according to incident CAN

The demographic and clinical characteristics of all subjects are shown in Table 1. The incidence of CAN was 10.6% (*n* = 53) over 2 years, and the mean age (SD) of all subjects was 58.5 (10.3) years. A total of 53 subjects had definite CAN, and one subject had concurrent orthostatic hypotension. Compared to the patients without CAN, those with CAN were more likely to have a long diabetic duration, high total cholesterol, and to use insulin. All baseline glycemic parameters such as GA, HbA1c, fasting plasma glucose (FPG), and postprandial glucose (PPG) were higher in subjects with CAN.

**Table 1:** Demographic and clinical variables at baseline according to the incidence of cardiovascular autonomic neuropathy

	Incident CAN		
	No (*n* = 445)	Yes (*n* = 53)	*P* value
Age (years)	58.5 ± 9.9	58.5 ± 13.0	0.999
Male, n (%)	273 (61.3)	26 (49.1)	0.084
Body mass index (kg/m^2^)	25.5 ± 6.8	25.6 ± 3.9	0.867
Duration of type 2 DM (years)	11.0 ± 6.8	15.2 ± 8.9	<0.001
Systolic BP (mmHg)	125.8 ± 14.3	128.5 ± 19.0	0.207
Diastolic BP (mmHg)	77.7 ± 10.5	75.5 ± 13.1	0.205
Lipid profile (mg/dL)			
Total cholesterol	143.6 ± 24.1	151.5 ± 23.5	0.025
Triglycerides	128.6 ± 68.4	139.0 ± 76.9	0.299
LDL cholesterol	78.1 ± 21.4	83.4 ± 20.6	0.715
HDL cholesterol	53.6 ± 14.7	52.8 ± 19.1	0.090
FPG (mg/dL)	137.1 ± 31.8	180.5 ± 76.9	<0.001
Postprandial glucose (mg/dL)^a^	195.3 ± 57.9	227.6 ± 74.9	<0.001
Glycated albumin (%)	16.9 ± 3.5	24.6 ± 7.8	<0.001
Hemoglobin A1c (%)	7.0 ± 0.8	8.7 ± 1.8	<0.001
Hemoglobin A1c (mmol/mol)	52.8 ± 9.3	71.7 ± 19.5	<0.001
Fasting C-peptide (ng/mL)	2.3 ± 1.1	2.0 ± 1.0	0.068
Estimated GFR (ml/min/1.73 m^2^)	84.5 ± 15.1	84.3 ± 16.9	0.933
Use of insulin, n (%)	51 (11.5)	25 (47.2)	<0.001
Use of oral anti-diabetic drug, n (%)			
Metformin, n (%)	406 (91.2)	47 (88.7)	0.540
Sulfonylurea, n (%)	138 (31.0)	15 (28.3)	0.687
Glinide, n (%)	3 (0.7)	2 (3.8)	0.032
Thiazolidinedione, n (%)	63 (14.2)	10 (18.9)	0.360
DPP-4 inhibitor, n (%)	263 (59.1)	33 (62.3)	0.658
α-Glucosidase inhibitor, n (%)	10 (2.2)	0 (0.0)	0.271
SGLT-2 inhibitor, n (%)	43 (9.7)	8 (15.1)	0.218
GLP-1 agonist	3 (0.7)	2 (3.8)	0.032
Use of lipid-lowering agent			
Statin, n (%)	319 (71.7)	38 (71.7)	0.841
Other, n (%)	8 (1.8)	0 (0.0)	0.318
Use of anti-hypertensive therapy			
ACE inhibitor or ARB, n (%)	166 (37.3)	27 (50.9)	0.054
CCB, n (%)	71 (16.0)	14 (26.4)	0.056
Thiazide, n (%)	52 (11.7)	7 (13.2)	0.746
Beta-blocker, n (%)	8 (1.8)	1 (1.9)	0.995
Current smoker, n (%)	99 (22.2)	12 (22.6)	0.948
Autonomic function test, n (%)			
Exhalation: inhalation ratio	1 (0.2)	8 (15.1)	<0.001
30:15 ratio	269 (60.4)	49 (92.5)	<0.001
Valsalva ratio	11 (2.5)	17 (32.1)	<0.001
Postural BP change	0 (0.0)	1 (1.9)	0.004

Data are presented as mean ± SD, median (25th to 75th percentile), or percentage

Lower limit of the age-specific reference range of the E:I ratio: age 20–24 years, 1.17; age 25–29, 1.15; age 30–34, 1.13; age 35–39, 1.12; age 40–44, 1.10; age 45–49, 1.08; age 50–54, 1.07; age 55–59, 1.06; age 60–64, 1.04; age 65–69, 1.03; and age 70–75, 1.02

Lower limit of the age-specific reference range of the 30:15 ratio: age 20–24 years 1.15; age 25–29, 1.14; age 30–34, 1.12; age 35–39, 1.11; age 40–44, 1.10; age 45–49, 1.09; age 50–54, 1.08; age 55–59, 1.07; age 60–64, 1.07; age 65–69, 1.06; and age 70–75, 1.06

Lower limit of the age-specific reference range of the Valsalva ratio: age 20–24 years, 1.43; age 25–29, 1.38; age 30–34, 1.33; age 35–39, 1.28; age 40–44, 1.24; age 45–49, 1.20; age 50–54, 1.16; age 55–59, 1.12; age 60–64, 1.08; age 65–69, 1.04; and age 70–75, 1.00

The reference range of postural BP change (decrease in systolic BP): normal ≤ 10 mmHg, borderline 11–29 mmHg, and abnormal ≥ 30 mmHg

*CAN* cardiovascular autonomic neuropathy, *DM* diabetes mellitus, *BP* blood pressure, *LDL*, low density lipoprotein, *HDL* high density lipoprotein, *FPG* fasting plasma glucose, *GFR* glomerular filtration rate, *DPP-4* dipeptidyl peptidase 4, *SGLT-2* sodium-glucose cotransporter 2, *GLP-1* glucagon-like peptide-1, *ACE* angiotensin-converting enzyme, *ARB* angiotensin receptor blocker, *CCB* calcium channel blocker

^a^Measured in 476 subjects

### Comparison of glycemic parameters over 2 years between patients with and without CAN

Both mean GA and mean HbA1c were significantly higher in subjects with CAN compared to subjects without it (Table 2). GA variability (SD of GA, adjusted SD of GA, and %CV of GA) and HbA1c variability (SD of HbA1c, adjusted SD of HbA1c, and %CV of HbA1c) were also significantly higher in subjects with CAN (Table 2). As shown in Additional file 2: Figure S2, mean GA was positively correlated with mean HbA1c (standardized β = 0.813, *P* < 0.001). The correlation between the %CV of GA and mean GA (standardized β = 0.452, *P* < 0.001) was more widely scattered than the correlation between the adjusted SD of GA and mean GA (standardized β = 0.725, *P* < 0.001) or between the %CV of HbA1c and mean HbA1c (standardized β = 0.578, *P* < 0.001).

**Table 2:** Comparison of glycemic parameters between patients with and without cardiovascular autonomic neuropathy over 2 years

	CAN (−)	CAN (+)	*P* value
Mean GA over 2 years (%)	17.0 ± 3.1	23.5 ± 6.8	<0.001
GA variability over 2 years			
Standard deviation of GA (%)	1.7 ± 1.1	4.6 ± 2.6	<0.001
Adjusted SD of GA (%)	1.6 ± 1.0	4.4 ± 2.4	<0.001
%CV of GA	9.7 ± 5.3	19.1 ± 8.3	<0.001
Measurement of GA, n	7.0 (6.0–8.0)	7.0 (7.0–8.0)	0.006
Mean HbA1c over 2 years (%)	7.0 ± 0.8	8.5 ± 1.4	<0.001
HbA1c variability over 2 years			
Standard deviation of HbA1c (%)	0.4 ± 0.3	0.9 ± 0.5	<0.001
Adjusted SD of HbA1c (%)	0.4 ± 0.3	0.9 ± 0.5	<0.001
%CV of HbA1c	6.0 ± 3.5	10.9 ± 4.9	<0.001
Measurement of HbA1c, n	8.0 (7.0–8.0)	8.0 (7.0–8.0)	0.056

Data are presented as mean ± SD or median (25th to 75th percentile)

Adjusted SD = SD/[n/(n − 1)]^0.5^, where n is the number of HbA1c or GA measurements

*CAN* cardiovascular autonomic neuropathy, *GA* glycated albumin, *HbA1c* hemoglobin A1c, *SD* standard deviation, *CV* coefficient of variance

### Effect of mean value versus GV parameters on CAN development

In univariate logistic regression analysis (Additional file 3: Table S1), duration of diabetes, FPG level, PPG level, baseline GA, baseline HbA1c, total cholesterol level, use of insulin, use of anti-hypertensive drugs, mean GA, parameters of GA variability, mean HbA1c, and parameters of HbA1c variability were significant risk factors of CAN development. All models were adjusted for duration of diabetes, total cholesterol level, use of insulin, use of anti-hypertensive drugs as significant risk factors in univariate analysis and for age [21], sex [21], fasting C-peptide level [22, 23] and smoking status [24] as known associated factors of CAN (Model 1–3, Table 3). When mean value of GA or HbA1c (Model 1), adjusted SD and %CV of GA or HbA1c (Model 2), or both mean values and GV parameters (Model 3) were added in multivariate logistic regression analyses, higher mean values and GV parameters produced significantly higher odds for CAN development (Table 3).

**Table 3:** Multivariate logistic regression analysis for the associations between glycemic parameters and cardiovascular autonomic neuropathy

	Model 1		Model 2		Model 3	
	OR (95% CI)	*P* value	OR (95% CI)	*P* value	OR (95% CI)	*P* value
Mean GA and GA variability over 2 years						
Mean GA (%)	1.35 (1.23–1.48)	<0.001			1.14 (1.02–1.28)	0.027
Adjusted SD of GA (%)			2.72 (2.06–3.58)	<0.001	2.23 (1.62–3.07)	<0.001
Mean GA (%)	1.35 (1.23–1.48)	<0.001			1.27 (1.15–1.41)	<0.001
%CV of GA			1.22 (1.15–1.30)	<0.001	1.18 (1.11–1.26)	<0.001
Mean HbA1c and HbA1c variability over 2 years						
Mean HbA1c (%)	3.21 (2.22–4.63)	<0.001			2.03 (1.29–3.20)	0.002
Adjusted SD of HbA1c (%)			20.20 (7.97–51.21)	<0.001	6.60 (2.05–21.24)	0.002
Mean HbA1c (%)	3.21 (2.22–4.63)	<0.001			2.36 (1.59–3.51)	<0.001
%CV of HbA1c			1.26 (1.17–1.36)	<0.001	1.17 (1.07–1.27)	<0.001

Adjusted SD = SD/[n/(n − 1)]^0.5^, where n is the number of HbA1c or GA measurements

All models were adjusted for age, sex, duration of diabetes, total cholesterol, fasting c-peptide, use of insulin, use of anti-hypertensive drugs, smoking status

Model 1 was additionally adjusted for mean value over 2 years

Model 2 was additionally adjusted for the parameters of glycemic variability

Model 3 was additionally adjusted for both mean value over 2 years and the parameters of glycemic variability

*OR* odds ratio, *CI* confidence interval, *SD* standard deviation, *CV* coefficient of variance

The AUCs for the prediction of CAN development did not differ between the mean values and GV parameters (mean GA vs. adjusted SD of GA, mean GA vs. %CV of GA, mean HbA1c vs. adjusted SD of HbA1c, and mean HbA1c vs. %CV of HbA1c), as listed in Additional file 3: Table S2.

### Comparison of predictive values for CAN Between GA- and HbA1c-related parameters

When each of the GA-related parameters and HbA1c-related parameters was respectively added in ROC analyses with and without adjustment for various covariates (Table 4), the AUCs of the mean GA, adjusted SD of GA, %CV of GA, mean HbA1c, adjusted SD of HbA1c, and %CV of HbA1c were all significant for predicting CAN. While the AUC for mean GA and the AUC for mean HbA1c were not significantly different, the AUC for the adjusted SD of GA was superior to the adjusted SD of HbA1c in predicting CAN, even after adjusting for age, sex, duration of diabetes, total cholesterol level, fasting C-peptide level, use of insulin, use of anti-hypertensive drugs, and smoking status (AUC for adjusted SD of GA = 0.876 vs. AUC for adjusted SD of HbA1c = 0.833, *P* = 0.013). The AUC for %CV of GA was also greater than that for the %CV of HbA1c in a multivariate model (AUC for %CV of GA = 0.865 vs. AUC for %CV of HbA1c = 0.822, *P* = 0.016).

**Table 4:** Comparison of predictive values for cardiovascular autonomic neuropathy between GA and HbA1c

	Crude model			Multivariate model^a^		
	Area under the curve	95% CI	*P* value	Area under the curve	95% CI	*P* value
Mean GA	0.831	0.768–0.895	<0.001	0.846	0.788–0.903	<0.001
Mean HbA1c	0.839	0.777–0.900	<0.001	0.824	0.757–0.892	<0.001
* P* value for comparison	0.759			0.212		
Adjusted SD of GA	0.877	0.828–0.925	<0.001	0.876	0.822–0.931	<0.001
Adjusted SD of HbA1c	0.835	0.776–0.893	<0.001	0.833	0.768–0.897	<0.001
* P *value for comparison	0.035			0.013		
%CV of GA	0.849	0.800–0.898	<0.001	0.865	0.814–0.916	<0.001
%CV of HbA1c	0.806	0.746–0.867	<0.001	0.822	0.760–0.885	<0.001
* P* value for comparison	0.043			0.016		

*CI* confidence interval, *SD* standard deviation, *CV* coefficient of variance

^a^Adjusted for age, sex, duration of diabetes, total cholesterol, fasting C-peptide, use of insulin, use of anti-hypertensive drugs, and smoking status

### The additive effect of each glycemic parameter for the prediction of CAN

**Fig. 1:**
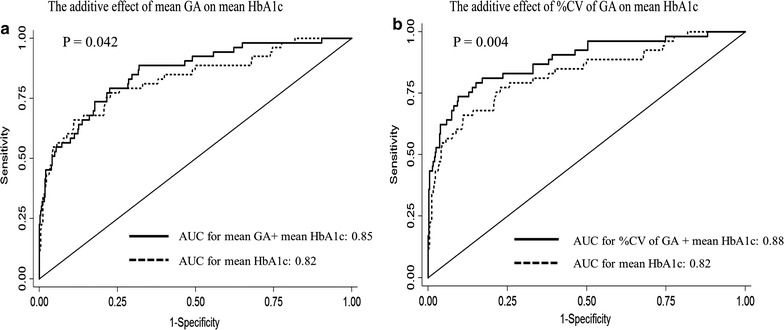
Receiver operating characteristic (ROC) curves indicating the additive effects of GA parameters on mean HbA1c. Two ROC curves were adjusted for age, sex, duration of diabetes, total cholesterol, fasting c-peptide, use of insulin, use of anti-hypertensive drugs, and smoking status. **a** Compares the predictive values between mean HbA1c (dashed line) and mean HbA1c plus mean GA (straight line). **b** Compares the predictive values between mean HbA1c (dashed line) and mean HbA1c plus %CV of GA (straight line)

Since GA-related parameters and HbA1c-related parameters showed similar predictive power for CAN development, the ROC curve evaluated the additive effects of GA on mean HbA1c; when mean GA level was added to mean HbA1c in multivariate logistic regression with adjustment for age, sex, duration of diabetes, total cholesterol level, fasting c-peptide level, use of insulin, use of anti-hypertensive drugs, and smoking status, the predictive value for CAN (AUC for mean GA and mean HbA1c together = 0.846 vs. AUC for mean HbA1c alone = 0.824, *P* = 0.042) was significantly improved compared to that of mean HbA1c alone (Fig. 1a). When the adjusted SD of GA (AUC for adjusted SD of GA and mean HbA1c together = 0.878 vs. AUC for mean HbA1c alone = 0.824, *P* = 0.007) or %CV of GA level was added to mean HbA1c in the same multivariate logstric regression model (AUC for %CV of GA and mean HbA1c together = 0.875 vs. AUC for mean HbA1c alone = 0.824, *P* = 0.004), the predictive value for CAN was also much improved compared to that of mean HbA1c alone (Fig. 1b).

Next, we compared the AUC of mean GA alone and the AUC of mean GA with adjusted SD of GA or %CV of GA to demonstrate the additive effect of GV on mean values (Additional file 3: Table S3). Whether or not other covariates were adjusted, addition of SD or %CV of GA enhanced the predictive power for CAN. When the AUC of mean GA with adjusted SD or %CV of GA was compared to the AUC of mean HbA1c with adjusted SD or CV of HbA1c, the AUCs of mean GA with GA variability remained superior to those of mean HbA1c with HbA1c variability in a multivariate model (Additional file 3: Table S3).

### Comparison of four groups classified by cut-off values of glycemic parameters

The cut-off value of mean HbA1c was 7.4% with 79.2% sensitivity and 80.7% specificity, that of mean GA was 18.5% with 77.4% sensitivity and 71.2% specificity, that of adjusted SD of HbA1c was 0.5% with 81.1% sensitivity and 78.0% specificity, that of adjusted SD of GA was 2.3% with 81.1% sensitivity and 80.7% specificity, that of %CV of HbA1c was 7.6 with 73.6% sensitivity and 76.4% specificity, and that of %CV of GA was 12.7 with 77.4% sensitivity and 75.5% specificity.

Based on these cut-off values, total subjects were assigned to four groups. Markedly higher ORs for CAN were observed in groups with high mean value (GA or HbA1c) and high GV parameter (adjusted SD or CV) in fully adjusted multivariate logistic models (Models A–E, Additional file 3: Table S4). High %CV of GA and high %CV of HbA1c had the highest ORs (Model F, Additional file 3: Table S4), and the same association was observed in the group with high adjusted SD of GA and high adjusted SD of HbA1c in a multivariate logistic analysis (data not shown).

## Discussion

This study demonstrated for the first time that 2-year consecutively measured GA level as a mean value and variability was significantly associated with CAN development, regardless of other conventional CVD risk factors. As in our previous study [13], this study consistently showed that mean glucose level and GV parameters were associated with CAN. However, we further evaluated a novel idea about the clinical utility of GA to predict CAN development compared to HbA1c; GA variability (SD and %CV) could more accurately predict CAN development than HbA1c variability, although the predictive value of mean GA did not differ from that of mean HbA1c. Each of mean GA, adjusted SD of GA, and %CV of GA level had distinct predictive value for CAN development and concurrently showed an additive effect for the prediction of CAN with mean HbA1c level. Furthermore, the predictive value of mean GA with GA variability was superior to that of mean HbA1c with HbA1c variability.

CAN is a common chronic diabetic complication that confers high mortality and morbidity in diabetic patients. Hypoglycemia unawareness promotes a reduced threshold for uncontrolled arrhythmia and sudden cardiac death [25]. Insensitivity to ischemic pain impairs the early recognition of myocardial infarction [26]. Imbalance between sympathetic and parasympathetic functions can deteriorate the autoregulation of cerebral blood flow and produce susceptibility to stroke [27]. CAN is closely associated with other diabetic microvascular complications such as retinopathy, nephropathy, and peripheral neuropathy, through changes in the vasomotor control of small vessels [28, 29]. Therefore, early detection and screening of patients with high risk for CAN development are clinically important to prevent potential progression to overt CVD and to identify concomitant diabetic complications.

The specific pathogenesis of CAN remains unclear. However, neuronal injury due to oxidative stress from poor glycemic control is the leading cause of the initiation of CAN development [18]. Sustained hyperglycemia induces the over-production of reactive oxygen species in the mitochondria, mitochondrial antioxidant enzymes such as superoxide dismutase 2 are depleted, and neuronal injury results [30]. Wide fluctuations in blood glucose level can trigger the same levels of oxidative stress as prolonged hyperglycemia. Postprandial hyperglycemia increased oxidative stress markers [31], and it was significantly higher in both the fasting state and the postprandial state in diabetic patients, whereas there was no postprandial elevation of oxidative stress markers in healthy subjects [32]. In our study, mean glucose level measures (GA and HbA1c) and their variability did not have a significant difference for predicting CAN development (Additional file 3: Table S2). Even the CVs of GA or HbA1c level, which adjusted the effect of mean glucose exposure, remained a significant risk factor for CAN. Therefore, the finding that the predictive power of GA variability for CAN development outperformed that of HbA1c variability was partially explained by GA’s shorter half-life, which enables it to reflect glucose excursions more accurately than does HbA1c [6, 33, 34]. The 2-year fluctuations in GA level were actually greater than those in HbA1c level in this study.

Together with being a marker of glucose control, GA might be a direct cause of diabetic complications including atherosclerosis [35]. GA is regarded as a precursor of advanced glycation end products (AGEs), which are thought to contribute to glucose toxicity [36]. AGEs result in increasing plasminogen activator inhibitor-1 and endothelin-1 production and impairment of endothelial nitric oxide (NO) synthase and NO actions, which can lead to reduction of neurovascular perfusion and cellular apoptosis [37]. GA accelerates the development and progression of atherosclerosis and vascular complications of diabetes via the proliferation and migration of vascular smooth muscle cells [38].

Although there has been no research evaluating the association between GA and CAN, growing evidence supports the crucial relationship between GA and subclinical or overt CVD; higher serum GA, rather than HbA1c, was significantly associated with more severe coronary artery stenosis [11, 39, 40]. Higher GA increased the risk for the presence of carotid plaque [12] and was positively related with carotid intima-media thickness [41, 42]. GA was associated with long-term cardiovascular outcomes including myocardial infarction, ischemic stroke, heart failure, and even death, independent of traditional CVD risk factors [43].

This study has several limitations. First, it has a retrospective design; thus, we cannot exclude the possibility of selection bias. Second, study subjects were recruited from a tertiary hospital and thus are not representative of the general Korean population. Third, concurrent hypoglycemia events were not assessed via continuous glucose monitoring, although hypoglycemia itself changes heart rate and blood pressure. However, there were no subjects who experienced a hypoglycemia event (FPG ≤ 70 mg/dL) during 2 years of observation, based on their consecutively measured FPG level in the outpatient clinic.

In conclusion, higher serum GA is significantly associated with higher risk of developing CAN. Mean GA and GA variability showed additive effects with mean HbA1c to improve the predictions for CAN, while each of them had a distinct predictive power to detect CAN. Although HbA1c has been accepted as a gold standard marker for monitoring glycemic status and diabetic complications, GA and its variability might be useful indicators for diabetic complications, and they also enhanced HbA1c’s modest clinical prediction of CAN development.

## Additional files

**Additional file 1: Figure S1.** Selection of enrolled subjects.

**Additional file 2: Figure S2.** Scatter plots for the correlations between mean value and GV parameters of GA and HbA1c. A Indicates a linear relationship between mean GA and mean HbA1c; B indicates a linear relationship between the adjusted SD of GA and mean GA. Adjusted SD means that the SD of GA was adjusted for the number of measurements. C Indicates a linear relationship between the %CV of GA and mean GA; D indicates a linear relationship between the adjusted SD of HbA1c and mean HbA1c. Adjusted SD means that the SD of HbA1c was adjusted for the number of measurements. E Indicates a linear relationship between the %CV of HbA1c and mean HbA1c.

**Additional file 3.** Supplementary tables.

## Abbreviations

CAN: cardiovascular autonomic neuropathy
CVD: cardiovascular disease
T2DM: type 2 diabetes mellitus
GA: glycated albumin
HbA1c: glycated hemoglobin
FPG: fasting plasma glucose
PPG: postprandial glucose
GV: glycemic variability
SD: standard deviation
CV: coefficient of variance
HRV: heart rate variability
AUC: area under the curve
ROC: receiver operating characteristic

## References

[1] Grundy SM, Benjamin IJ, Burke GL, Chait A, Eckel RH, Howard BV, Mitch W, Smith SC, Sowers JR (1999). Diabetes and cardiovascular disease: a statement for healthcare professionals from the American Heart Association. Circulation.

[2] Pop-Busui R (2010). Cardiac autonomic neuropathy in diabetes: a clinical perspective. Diabetes Care.

[3] Ceriello A, Esposito K, Piconi L, Ihnat MA, Thorpe JE, Testa R, Boemi M, Giugliano D (2008). Oscillating glucose is more deleterious to endothelial function and oxidative stress than mean glucose in normal and type 2 diabetic patients. Diabetes.

[4] Lee JE (2015). Alternative biomarkers for assessing glycemic control in diabetes: fructosamine, glycated albumin, and 1,5-anhydroglucitol. Ann Pediatr Endocrinol Metab.

[5] Koga M (2014). Glycated albumin; clinical usefulness. Clin Chimica Acta Int J Clin Chem.

[6] Koga M, Murai J, Morita S, Saito H, Kasayama S (2013). Comparison of annual variability in HbA1c and glycated albumin in patients with type 1 vs. type 2 diabetes mellitus. J Diabetes Complicat.

[7] Hirsch IB, Brownlee M (2010). Beyond hemoglobin A1c–need for additional markers of risk for diabetic microvascular complications. JAMA.

[8] Pan J, Li Q, Zhang L, Jia L, Tang J, Bao Y, Jia W (2014). Serum glycated albumin predicts the progression of diabetic retinopathy—a five year retrospective longitudinal study. J Diabetes Complicat.

[9] Selvin E, Rawlings AM, Grams M, Klein R, Sharrett AR, Steffes M, Coresh J (2014). Fructosamine and glycated albumin for risk stratification and prediction of incident diabetes and microvascular complications: a prospective cohort analysis of the Atherosclerosis Risk in Communities (ARIC) study. Lancet Diabetes Endocrinol.

[10] Wang N, Guo C, Han P, Li T (2016). Glycated albumin indicates peripheral diabetic neuropathy. Acta Diabetol.

[11] Pu LJ, Lu L, Shen WF, Zhang Q, Zhang RY, Zhang JS, Hu J, Yang ZK, Ding FH, Chen QJ (2007). Increased serum glycated albumin level is associated with the presence and severity of coronary artery disease in type 2 diabetic patients. Circ J Off J Japn Circ Soc.

[12] Sato Y, Nagao M, Asai A, Nakajima Y, Takaya M, Takeichi N, Takemitsu S, Sudo M, Kano-Wakakuri T, Ishizaki A (2013). Association of glycated albumin with the presence of carotid plaque in patients with type 2 diabetes. J Diabetes Investig.

[13] Jun JE, Jin SM, Baek J, Oh S, Hur KY, Lee MS, Lee MK, Kim JH (2015). The association between glycemic variability and diabetic cardiovascular autonomic neuropathy in patients with type 2 diabetes. Cardiovasc Diabetol.

[14] Penno G, Solini A, Bonora E, Fondelli C, Orsi E, Zerbini G, Morano S, Cavalot F, Lamacchia O, Laviola L (2013). HbA1c variability as an independent correlate of nephropathy, but not retinopathy, in patients with type 2 diabetes: the Renal Insufficiency and Cardiovascular Events (RIACE) Italian multicenter study. Diabetes Care.

[15] Hermann JM, Hammes HP, Rami-Merhar B, Rosenbauer J, Schutt M, Siegel E, Holl RW (2014). HbA1c variability as an independent risk factor for diabetic retinopathy in type 1 diabetes: a German/Austrian multicenter analysis on 35,891 patients. PLoS ONE.

[16] Kilpatrick ES, Rigby AS, Atkin SL (2008). A1C variability and the risk of microvascular complications in type 1 diabetes: data from the Diabetes Control and Complications Trial. Diabetes Care.

[17] Karayannis G, Giamouzis G, Cokkinos DV, Skoularigis J, Triposkiadis F (2012). Diabetic cardiovascular autonomic neuropathy: clinical implications. Exp Rev Cardiovasc Ther.

[18] Dimitropoulos G, Tahrani AA, Stevens MJ (2014). Cardiac autonomic neuropathy in patients with diabetes mellitus. World J Diabetes.

[19] Balcioglu AS, Muderrisoglu H (2015). Diabetes and cardiac autonomic neuropathy: clinical manifestations, cardiovascular consequences, diagnosis and treatment. World J Diabetes.

[20] Spallone V, Ziegler D, Freeman R, Bernardi L, Frontoni S, Pop-Busui R, Stevens M, Kempler P, Hilsted J, Tesfaye S (2011). Cardiovascular autonomic neuropathy in diabetes: clinical impact, assessment, diagnosis, and management. Diabetes Metab Res Rev.

[21] Rolim LC, Sa JR, Chacra AR, Dib SA (2008). Diabetic cardiovascular autonomic neuropathy: risk factors, clinical impact and early diagnosis. Arq Bras Cardiol.

[22] Gottsater A, Ahmed M, Fernlund P, Sundkvist G (1999). Autonomic neuropathy in Type 2 diabetic patients is associated with hyperinsulinaemia and hypertriglyceridaemia. Diabetic Med J Br Diabet Assoc.

[23] Toyry JP, Niskanen LK, Mantysaari MJ, Lansimies EA, Uusitupa MI (1996). Occurrence, predictors, and clinical significance of autonomic neuropathy in NIDDM. Ten-year follow-up from the diagnosis. Diabetes.

[24] Middlekauff HR, Park J, Moheimani RS (2014). Adverse effects of cigarette and noncigarette smoke exposure on the autonomic nervous system: mechanisms and implications for cardiovascular risk. J Am Coll Cardiol.

[25] Snell-Bergeon JK, Wadwa RP (2012). Hypoglycemia, diabetes, and cardiovascular disease. Diabetes Technol Thera.

[26] Young LH, Wackers FJ, Chyun DA, Davey JA, Barrett EJ, Taillefer R, Heller GV, Iskandrian AE, Wittlin SD, Filipchuk N (2009). Cardiac outcomes after screening for asymptomatic coronary artery disease in patients with type 2 diabetes: the DIAD study: a randomized controlled trial. JAMA.

[27] Marthol H, Brown CM, Zikeli U, Ziegler D, Dimitrov N, Baltadzhieva R, Hilz MJ (2006). Altered cerebral regulation in type 2 diabetic patients with cardiac autonomic neuropathy. Diabetologia.

[28] Valensi P, Paries J, Attali JR (2003). Cardiac autonomic neuropathy in diabetic patients: influence of diabetes duration, obesity, and microangiopathic complications—the French multicenter study. Metab Clin Exp.

[29] Motataianu A, Balasa R, Voidazan S, Bajko Z (2013). Cardiovascular autonomic neuropathy in context of other complications of type 2 diabetes mellitus. Biomed Res Int.

[30] Sharma R, Buras E, Terashima T, Serrano F, Massaad CA, Hu L, Bitner B, Inoue T, Chan L, Pautler RG (2010). Hyperglycemia induces oxidative stress and impairs axonal transport rates in mice. PLoS ONE.

[31] Wright E, Scism-Bacon JL, Glass LC (2006). Oxidative stress in type 2 diabetes: the role of fasting and postprandial glycaemia. Int J Clin Pract.

[32] Ceriello A, Quagliaro L, Catone B, Pascon R, Piazzola M, Bais B, Marra G, Tonutti L, Taboga C, Motz E (2002). Role of hyperglycemia in nitrotyrosine postprandial generation. Diabetes Care.

[33] Hayashi A, Takano K, Masaki T, Yoshino S, Ogawa A, Shichiri M (2016). Distinct biomarker roles for HbA1c and glycated albumin in patients with type 2 diabetes on hemodialysis. J Diabetes Complic.

[34] Suwa T, Ohta A, Matsui T, Koganei R, Kato H, Kawata T, Sada Y, Ishii S, Kondo A, Murakami K (2010). Relationship between clinical markers of glycemia and glucose excursion evaluated by continuous glucose monitoring (CGM). Endocr J.

[35] Arasteh A, Farahi S, Habibi-Rezaei M, Moosavi-Movahedi AA (2014). Glycated albumin: an overview of the in vitro models of an in vivo potential disease marker. J Diabetes Metab Disord.

[36] Rodino-Janeiro BK, Gonzalez-Peteiro M, Ucieda-Somoza R, Gonzalez-Juanatey JR, Alvarez E (2010). Glycated albumin, a precursor of advanced glycation end-products, up-regulates NADPH oxidase and enhances oxidative stress in human endothelial cells: molecular correlate of diabetic vasculopathy. Diabetes Metab Res Rev.

[37] Giacco F, Brownlee M (2010). Oxidative stress and diabetic complications. Circ Res.

[38] Hattori Y, Suzuki M, Hattori S, Kasai K (2002). Vascular smooth muscle cell activation by glycated albumin (Amadori adducts). Hypertension..

[39] Ma X, Hu X, Zhou J, Hao Y, Luo Y, Lu Z, Bao Y, Jia W (2015). Glycated albumin is more closely correlated with coronary artery disease than 1,5-anhydroglucitol and glycated hemoglobin A1c. Cardiovasc Diabetol.

[40] Shen Y, Pu LJ, Lu L, Zhang Q, Zhang RY, Shen WF (2012). Glycated albumin is superior to hemoglobin A1c for evaluating the presence and severity of coronary artery disease in type 2 diabetic patients. Cardiology.

[41] Mukai N, Ninomiya T, Hata J, Hirakawa Y, Ikeda F, Fukuhara M, Hotta T, Koga M, Nakamura U, Kang D (2015). Association of hemoglobin A1c and glycated albumin with carotid atherosclerosis in community-dwelling Japanese subjects: the Hisayama Study. Cardiovasc Diabetol.

[42] Furusyo N, Koga T, Ai M, Otokozawa S, Kohzuma T, Ikezaki H, Schaefer EJ, Hayashi J (2013). Plasma glycated albumin level and atherosclerosis: results from the Kyushu and Okinawa Population Study (KOPS). Int J Cardiol.

[43] Selvin E, Rawlings AM, Lutsey PL, Maruthur N, Pankow JS, Steffes M, Coresh J (2015). Fructosamine and glycated albumin and the risk of cardiovascular outcomes and death. Circulation.

