# Supplementary figures and images for: Glycated albumin and its variability as an indicator of cardiovascular autonomic neuropathy development in type 2 diabetic patients
Source: Cardiovasc Diabetol. 2017 Oct 10;16:127. doi: 10.1186/s12933-017-0619-2 (PMC5635541; doi:10.1186/s12933-017-0619-2)

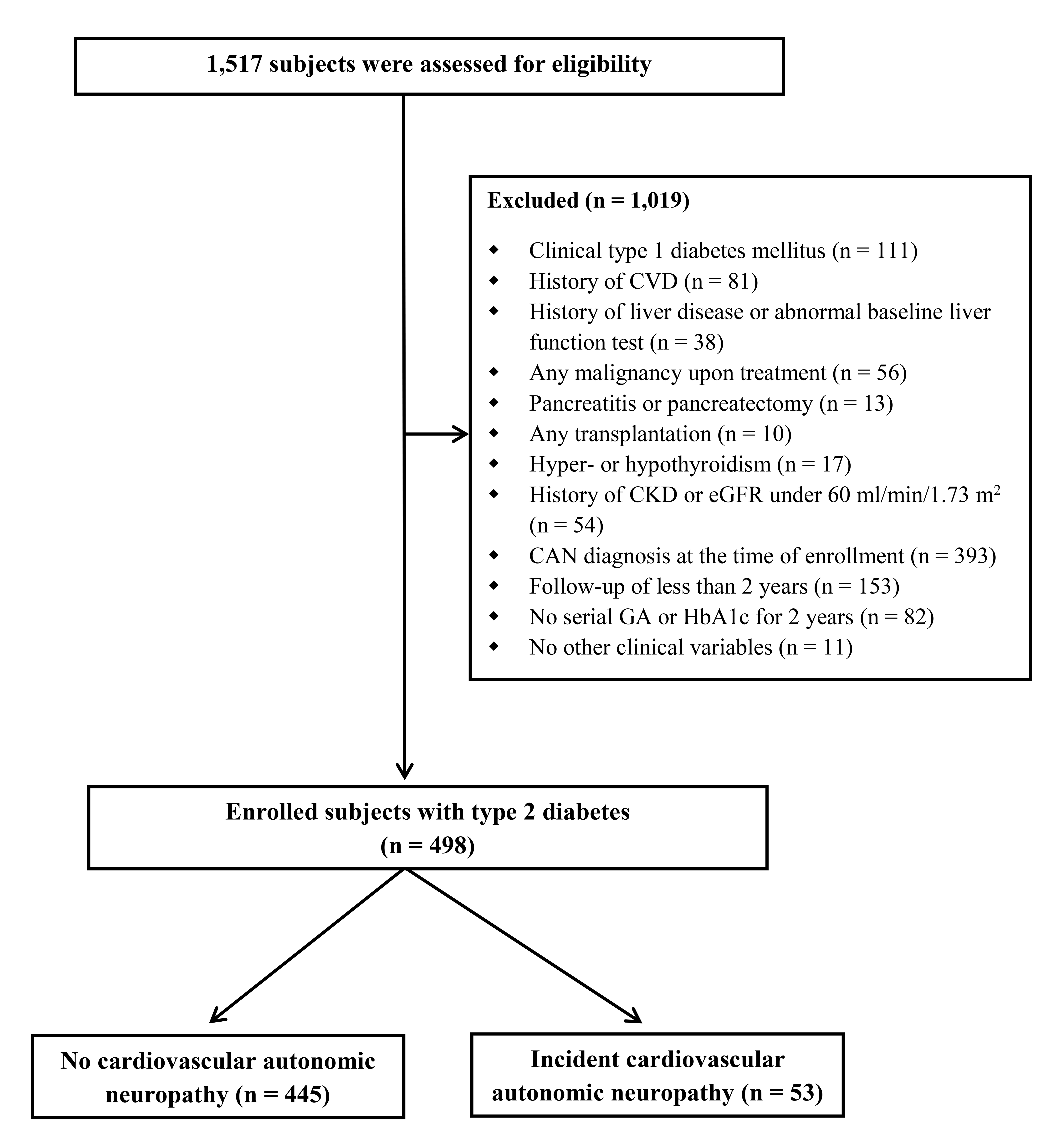

Supplement: Supplementary file 1 — Additional file 1: Figure S1. Selection of enrolled subjects. [file 12933_2017_619_MOESM1_ESM.tif]

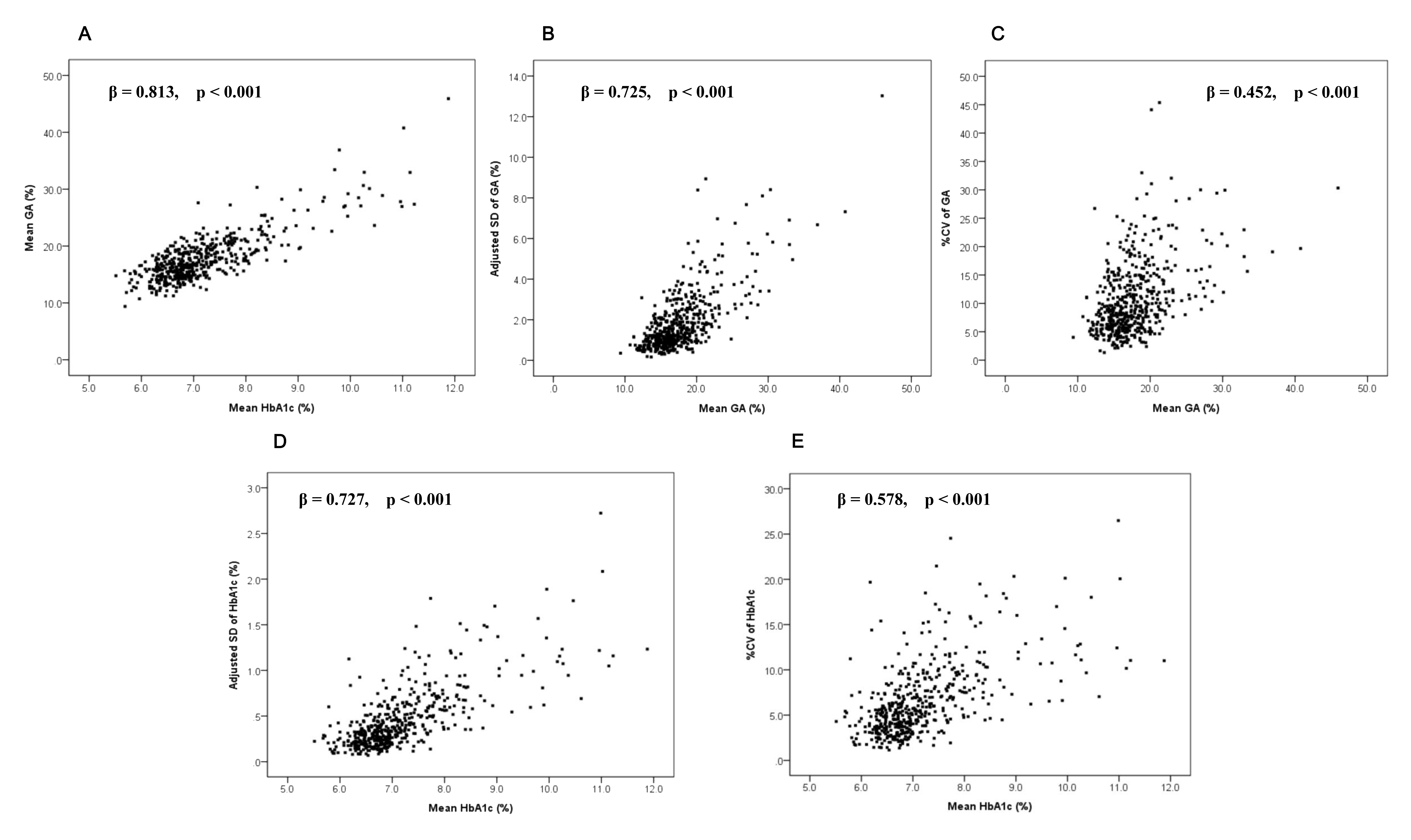

Supplement: Supplementary file 2 — Additional file 2: Figure S2. Scatter plots for the correlations between mean value and GV parameters of GA and HbA1c. A Indicates a linear relationship between mean GA and mean HbA1c; B indicates a linear relationship between the adjusted SD of GA and mean GA. Adjusted SD means that the SD of GA was adjusted for the number of measurements. C Indicates a linear relationship between the %CV of GA and mean GA; D indicates a linear relationship between the adjusted SD of HbA1c and mean HbA1c. Adjusted SD means that the SD of HbA1c was adjusted for the number of measurements. E Indicates a linear relationship between the %CV of HbA1c and mean HbA1c. [file 12933_2017_619_MOESM2_ESM.tif]
