# Supplementary material for: Glycated albumin and its variability as an indicator of cardiovascular autonomic neuropathy development in type 2 diabetic patients
Source: Cardiovasc Diabetol. 2017 Oct 10;16:127. doi: 10.1186/s12933-017-0619-2 (PMC5635541; doi:10.1186/s12933-017-0619-2)
Supplement: Supplementary file 3 — Additional file 3. Supplementary tables. [file 12933_2017_619_MOESM3_ESM.docx]

**Supplementary Table 1. Univariate logistic regression analysis for the risk of cardiovascular autonomic neuropathy**

| **Variables** | **Univariate OR (95% CI)** | ***P*-value** |
| --- | --- | --- |
| Age (years) | 1.00 (0.97-1.03) | 0.999 |
| Male | 0.61 (0.34-1.07) | 0.086 |
| Body mass index (kg/m^2^) | 1.00 (0.97-1.04) | 0.867 |
| Duration of type 2 DM (per 10 years) | 2.00 (1.41-2.85) | **<0.001** |
| Systolic blood pressure (mmHg) | 1.01 (0.99-1.03) | 0.207 |
| Diastolic blood pressure (mmHg) | 0.98 (0.96-1.01) | 0.205 |
| Lipid profile (mg/dL) |  |  |
| Total cholesterol | 1.01 (1.00-1.03) | **0.026** |
| Triglycerides | 1.00 (0.99-1.01) | 0.300 |
| HDL cholesterol | 0.99 (0.98-1.02) | 0.714 |
| LDL cholesterol | 1.01 (0.99-1.02) | 0.091 |
| Fasting plasma glucose (mg/dL) | 1.02 (1.01-1.03) | **<0.001** |
| Postprandial glucose (mg/dL) | 1.01 (1.00-1.02) | **<0.001** |
| Glycated albumin (%) | 1.32 (1.23-1.41) | **<0.001** |
| Hemoglobin A1c (%) | 2.75 (2.12-3.59) | **<0.001** |
| Fasting C-peptide (ng/mL) | 0.76 (0.57-1.02) | 0.068 |
| Estimated GFR (ml/min/1.73 m^2^) | 1.00 (0.98-1.02) | 0.933 |
| Use of insulin (yes) | 6.90 (3.74-12.74) | **<0.001** |
| Use of anti-hypertensive drugs (yes) | 1.79 (1.01-3.19 ) | **0.048** |
| Use of lipid-lowering drugs (yes) | 0.85 (0.47-1.55) | 0.599 |
| Current smoker | 1.02 (0.52-2.02) | 0.948 |
| Mean GA over 2 years (%) | 1.37 (1.27-1.48) | **<0.001** |
| GA variability over 2 years |  |  |
| SD of GA (%) | 2.47 (1.99-3.06) | **<0.001** |
| Adjusted SD of GA (%) | 2.67 (2.11-3.37) | **<0.001** |
| %CV of GA | 1.21 (1.16-1.27) | **<0.001** |
| Mean HbA1c over 2 years (%) | 3.31 (2.46-4.46) | **<0.001** |
| HbA1c variability over 2 years |  |  |
| SD of HbA1c (%) | 17.34 (8.17-36.95) | **<0.001** |
| Adjusted SD of HbA1c (%) | 21.95 (9.72-49.56) | **<0.001** |
| %CV of HbA1c | 1.26 (1.18-1.35) | **<0.001** |

OR, odds ratio; CI, confidence interval; CAN, cardiovascular autonomic neuropathy; DM, diabetes mellitus; HDL, high-density lipoprotein; LDL, low-density lipoprotein; GFR, glomerular filtration rate; SD, standard deviation; CV, coefficient of variance

**Supplementary Table 2. The comparison of predictive values for cardiovascular autonomic neuropathy between mean glycemic value and parameters of glycemic variability**

|  | **Crude model** | |  | **Multivariate model*** | |
| --- | --- | --- | --- | --- | --- |
|  | Area under the curve | 95% CI |  | Area under the curve | 95% CI |
| Mean GA | 0.831 | 0.768-0.895 |  | 0.846 | 0.788-0.903 |
| Adjusted SD of GA | 0.877 | 0.828-0.925 |  | 0.876 | 0.822-0.931 |
| *P* value for comparison | 0.097 | |  | 0.113 | |
| Mean GA | 0.831 | 0.768-0.895 |  | 0.846 | 0.788-0.903 |
| %CV of GA | 0.849 | 0.800-0.898 |  | 0.865 | 0.814-0.916 |
| *P* value for comparison | 0.614 | |  | 0.367 | |
| Mean HbA1c | 0.839 | 0.777-0.900 |  | 0.824 | 0.757-0.892 |
| Adjusted SD of HbA1c | 0.835 | 0.776-0.893 |  | 0.833 | 0.768-0.897 |
| *P*-value for comparison | 0.871 | |  | 0.602 | |
| Mean HbA1c | 0.839 | 0.777-0.900 |  | 0.824 | 0.757-0.892 |
| %CV of HbA1c | 0.806 | 0.746-0.867 |  | 0.822 | 0.760-0.885 |
| *P*-value for comparison | 0.281 | |  | 0.903 | |

*****Adjusted for age, sex, duration of diabetes, total cholesterol, fasting c-peptide, use of insulin, use of anti-hypertensive drugs, and smoking status

CI, confidence interval; SD, standard deviation; CV, coefficient of variance

**Supplementary Table 3. The additive effects of parameters of glycemic variability on mean values**

|  | **Crude model** | | |  | **Multivariate model*** | |
| --- | --- | --- | --- | --- | --- | --- |
|  | Area under the curve | | 95% CI |  | Area under the curve | 95% CI |
| Mean GA | | 0.831 | 0.768-0.895 |  | 0.846 | 0.788-0.903 |
| Mean GA + Adjusted SD of GA | | 0.881 | 0.829-0.933 |  | 0.882 | 0.828-0.936 |
| *P* value for comparison | | **0.003** | |  | **0.014** | |
| Mean GA | | 0.831 | 0.768-0.895 |  | 0.846 | 0.788-0.903 |
| Mean GA+ %CV of GA | | 0.883 | 0.831-0.934 |  | 0.886 | 0.833-0.938 |
| *P* value for comparison | | **0.003** | |  | **0.007** | |
| Mean GA + Adjusted SD of GA | | 0.881 | 0.829-0.933 |  | 0.882 | 0.828-0.936 |
| Mean HbA1c + Adjusted SD of HbA1c | | 0.853 | 0.795-0.911 |  | 0.842 | 0.775-0.908 |
| *P*-value for comparison | | 0.189 | |  | **0.021** | |
| Mean GA + %CV of GA | | 0.883 | 0.831-0.934 |  | 0.886 | 0.833-0.938 |
| Mean HbA1c + %CV of HbA1c | | 0.853 | 0.795-0.912 |  | 0.844 | 0.779-0.910 |
| *P*-value for comparison | | 0.142 | |  | **0.018** | |

*****Adjusted for age, sex, duration of diabetes, total cholesterol, fasting c-peptide, use of insulin, use of anti-hypertensive drugs, and smoking status

CI, confidence interval; SD, standard deviation; CV, coefficient of variance

**Supplementary Table 4. Adjusted odds ratios for CAN in four different groups according to cut-off values of GA- and HbA1c-related parameters**

| **A. Mean GA and adjusted SD of GA** | | Adjusted OR  (95% CI) | *P*-value | **B. Mean GA and %CV of GA** | Adjusted OR (95% CI) | *P*-value |
| --- | --- | --- | --- | --- | --- | --- |
| Mean GA <18.5% + Adjusted SD of GA <2.3% | | Reference |  | Mean GA <18.5% + %CV of GA <12.7 | Reference |  |
| Mean GA ≥18.5% + Adjusted SD of GA <2.3% | | 7.55 (2.12-26.92) | 0.002 | Mean GA ≥18.5% + %CV of GA <12.7 | 4.61 (1.33-16.02) | 0.016 |
| Mean GA <18.5% + Adjusted SD of GA ≥2.3% | | 2.90 (0.73-11.59) | 0.132 | Mean GA <18.5% + %CV of GA ≥12.7 | 5.52 (1.58-19.36) | 0.009 |
| Mean GA ≥18.5% + Adjusted SD of GA ≥2.3% | | 29.43 (9.73-88.98) | <0.001 | Mean GA ≥18.5% + %CV of GA ≥12.7 | 22.72 (7.62-67.75) | <0.001 |
| *P* for trend | | <0.001 |  | *P* for trend | <0.001 |  |
| **C. Mean HbA1c and %CV of HbA1c** | | Adjusted OR  (95% CI) | *P*-value | **D. Mean HbA1c and mean GA** | Adjusted OR (95% CI) | *P*-value |
| Mean HbA1c <7.4% + %CV of HbA1c <7.6 | Reference | |  | Mean HbA1c <7.4% + Mean GA <18.5% | Reference |  |
| Mean HbA1c ≥7.4% + %CV of HbA1c <7.6 | 6.94 (2.05-23.45) | | 0.002 | Mean HbA1c ≥7.4% + Mean GA <18.5% | 2.02 (0.55-7.49) | 0.291 |
| Mean HbA1c <7.4% + %CV of HbA1c ≥7.6 | 4.29 (1.27-14.47) | | 0.019 | Mean HbA1c <7.4% + Mean GA ≥18.5% | 4.26 (1.12-16.14) | 0.033 |
| Mean HbA1c ≥7.4% + %CV of HbA1c ≥7.6 | 22.10 (8.29-58.95) | | <0.001 | Mean HbA1c ≥7.4% + Mean GA ≥18.5% | 19.06 (7.19-50.55) | <0.001 |
| *P* for trend | <0.001 | |  | *P* for trend | <0.001 |  |
| **E. Mean HbA1c and %CV of GA** | Adjusted OR  (95% CI) | | *P*-value | **F. %CV of HbA1c and %CV of GA** | Adjusted OR (95% CI) | *P*-value |
| Mean HbA1c <7.4% + %CV of GA <12.7 | Reference | |  | %CV of HbA1c <7.6 + %CV of GA <12.7 | Reference |  |
| Mean HbA1c ≥7.4% + %CV of GA <12.7 | 8.99 (2.28-35.47) | | 0.002 | %CV of HbA1c ≥7.6 + %CV of GA <12.7 | 2.82 (0.67-11.91) | 0.158 |
| Mean HbA1c <7.4% + %CV of GA ≥12.7 | 9.85 (2.77-35.00) | | <0.001 | %CV of HbA1c <7.6 + %CV of GA ≥12.7 | 6.96 (2.17-22.33) | 0.001 |
| Mean HbA1c ≥7.4% + %CV of GA ≥12.7 | 54.04 (16.09-181.56) | | <0.001 | %CV of HbA1c ≥7.6 + %CV of GA ≥12.7 | 15.10 (6.26-36.42) | <0.001 |
| *P* for trend | <0.001 | |  | *P* for trend | <0.001 |  |

All models were adjusted for age, sex, duration of diabetes, total cholesterol, fasting c-peptide, use of insulin, use of anti-hypertensive drugs, and smoking status.
